# Absolute Configuration Determination of Two Diastereomeric Neovasifuranones A and B from *Fusarium oxysporum* R1 by a Combination of Mosher’s Method and Chiroptical Approach

**DOI:** 10.3390/jof8010040

**Published:** 2021-12-31

**Authors:** Zhiyang Fu, Yuanyuan Liu, Meijie Xu, Xiaojun Yao, Hong Wang, Huawei Zhang

**Affiliations:** 1 School of Pharmaceutical Sciences, Zhejiang University of Technology, Hangzhou 310014, China; fuzhiyoung@163.com (Z.F.); liuyuan_0507@163.com (Y.L.); xumeijie1230@163.com (M.X.); hongw@zjut.edu.cn (H.W.); 2 Department of Chemistry and Chemical Engineering, Lanzhou University, Lanzhou 730000, China; xjyao@lzu.edu.cn; 3 Key Laboratory of Marine Fishery Resources Exploitment & Utilization of Zhejiang Province, Hangzhou 310014, China

**Keywords:** endophytic fungus, *Fusarium oxysporum*, secondary metabolite, absolute configuration, chiroptical method, Mosher’s reaction

## Abstract

Endophytic fungi are one of prolific sources of bioactive natural products with potential application in biomedicine and agriculture. In our continuous search for antimicrobial secondary metabolites from *Fusarium oxysporum* R1 associated with traditional Chinese medicinal plant *Rumex madaio* Makino using one strain many compounds (OSMAC) strategy, two diastereomeric polyketides neovasifuranones A (**3**) and B (**4**) were obtained from its solid rice medium together with *N*-(2-phenylethyl)acetamide (**1**), 1-(3-hydroxy-2-methoxyphenyl)-ethanone (**2**) and 1,2-*seco*-trypacidin (**5**). Their planar structures were unambiguously determined using 1D NMR and MS spectroscopy techniques as well as comparison with the literature data. By a combination of the modified Mosher’s reactions and chiroptical methods using time-dependent density functional theory-electronic circular dichroism (TDDFT-ECD) and optical rotatory dispersion (ORD), the absolute configurations of compounds **3** and **4** are firstly confirmed and, respectively, characterized as (4*S*,7*S*,8*R)*, (4*S*,7*S*,8*S)*. Bioassay results indicate that these metabolites **1**–**5** exhibit weak inhibitory effect on *Helicobacter pylori* 159 with MIC values of ≥16 μg/mL. An in-depth discussion for enhancement of fungal metabolite diversity is also proposed in this work.

## 1. Introduction

**Figure 1:**
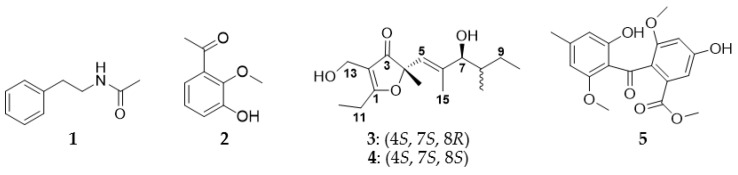
Chemical structures of compounds **1**–**5** from *Fusarium oxysporum* R1.

The assignment of absolute configuration (AC) is one of the most challenging tasks in the structure elucidation of chiral natural products. Application of Mosher’s method or quantum mechanical calculation of chiroptical properties had proved to be practical and reliable, including time-dependent density functional theory-electronic circular dichroism (TDDFT-ECD) and optical rotatory dispersion (ORD) [1,2,3,4]. However, some difficulties and uncertainties still exist in determining ACs of molecules with high conformational flexibility using single method. Therefore, a combined application of these approaches is necessary and has been shown to be valid in some cases, such as chenopodolans B and D [5,6], sapinofuranones B and C [7] and ent-thailandolide B [8]. A growing number of evidence indicates that the genus *Fusarium* is one rich source of secondary metabolites with a wide variety of chemical structures and biological properties [9]. In our continuous search for antimicrobial secondary metabolites from the endophytic strain *F. oxysporum* R1 associated with traditional Chinese medicinal plant *Rumex madaio* Makino using one strain many compounds (OSMAC) strategy [10,11], chemical study of the ethyl acetate extract of its rice medium resulted in the isolation of five known compounds including *N*-(2-phenylethyl)acetamide (**1**), 1-(3-hydroxy-2-methoxyphenyl)-ethanone (**2**), neovasifuranones A (**3**) and B (**4**) and 1,2-*seco*-trypacidin (**5**) (Figure 1). Compounds **3** and **4** are diastereomeric polyketides originally isolated from the phytopathogenic strain *Neocosmospora vasinfecta* NHL2298 [12,13] and later found to be produced by the soil-derived strain *Penicillium* sp. SYPF7381 [14] and the endophytic fungus *Aspergillus japonicus* CAM231 from *Garcinia preussii* [15]. However, their ACs are still unassigned. Herein the present work highlights on assignment of ACs in **3** and **4** by a combined application of Mosher’s method and quantum mechanical calculation of chiroptical (ECD and ORD) properties.

## 2. Materials and Methods

### 2.1. General

The NMR spectra were determined on Bruker Avance DRX600 instruments (600 MHz for ^1^H and 150.92 MHz for ^13^C NMR) (Bruker, Fällande, Switzerland). ESIMS were obtained with an Agilent 6210 LC/TOF-MS spectrometer (Agilent Technologies, Santa Clara, CA, USA). Optical rotation and CD spectra were performed on JASCO P-2000 polarimeter and JASCO J-1500 spectrometer (JASCO, Fukuoka, Japan). UV and IR spectra were measured through a Hitachi-UV-3000 spectrometer (Hitachi, Tokyo, Japan) and a Nexus 870 spectrometer (Thermo-Nicolet, Madison, WI, USA), respectively. Reverse phase HPLC was carried out on an Essentia LC-16P apparatus (Essentia, San Diego, CA, USA) fitted with a preparative HPLC column (Phenomenex Gemini-NX C18, 50 mm £ 21.2 mm, 5 mm) or a semi-preparative column (Phenomenex Synergi Hydro-RP, 250 × 10 mm, 4 µm). Acetonitrile and H_2_O used in HPLC system were chromatographic grade, and all other chemicals were analytical.

### 2.2. Biological Material

The endophytic fungal strain R1 was isolated from the healthy plant *R. madaio* Makino collected off the coastal region of Putuo Island, China [16], and molecularly identified as *F. oxysporum* according to its 18S rDNA gene sequence (GenBank accession No. MF376147) and deposited at China General Microbiological Culture Collection Centre (CGMCC no. 17763) [11].

### 2.3. Fermentation, Extraction and Isolation

The strain R1 grown on potato dextrose agar (PDA) media was inoculated into 500 mL Erlenmeyer flasks containing 200 mL potato dextrose broth (PDB) medium, and shaken for 3 days at 200 rpm and 30 °C. The fermentation was performed in Erlenmeyer flasks (50 × 1 L) with sterilized rice (160 g) and tap water (320 mL). After autoclaving at 121 °C for 20 min, each flask was inoculated with 5% seed cultures and then incubated at room temperature under static conditions for 30 days. The fermented rice of each flask was extracted with 500 mL EtOAc by an ultrasonic instrument for 20 min, 3 times followed by filtration using gauze. All filtrate was combined and evaporated under vacuum to dryness, affording the crude extract (approximate 19 g). Then the extract was quickly separated using HPLC on a preparative column to afford six fractions A-F, and further purified using a semi-preparative column for subdivision [17]. Compound **1** (1.8 mg, t_R_ = 7.2 min) and compound **2** (1.8 mg, t_R_ = 8.2 min) were obtained from fraction A with 30% CH_3_CN/H_2_O with a flow rate of 3.0 mL/min at 210 nm. Compound **3** (23.4 mg, t_R_ = 9.0 min) and compound **4** (8.2 mg, t_R_ = 10.5 min) were isolated from fraction C with 40% CH_3_CN/H_2_O. Compound **5** (4.6 mg, t_R_ = 10.7 min) was purified from fraction D with 50% CH_3_CN/H_2_O.

### 2.4. Preparation of (R)- and (S)-MTPA Esters of Compounds **3** and **4**

Compound **3** (1.2 mg, 4.26 μmol) was transferred into a NMR tube and dried under vacuum. Pyridine-*d*_5_ (0.5 mL) and (*S*)-(-)-α-methoxy-α-(trifluoromethyl)phenylacetyl chloride (5 μL, 26.5 μmol) were added under a N_2_ gas stream, and the NMR tube was shaken carefully to mix the sample and the MTPA chloride. The acylation was achieved at 20 °C for 36 h [18], suggesting that compound **3** was entirely transformed into the desired product (*R*)-MTPA ester derivative **3a**: ^1^H NMR (600 MHz, pyridine-*d*_5_) δ_H_ 5.902 (1H, d, H-5), 5.404 (1H, d, H-7), 1.322 (1H, m, H-8), 1.177 (1H, m, H-9a), 1.291 (1H, m, H-9b), 1.541 (3H, s, H-14), 1.847 (3H, s, H-15), 1.167 (3H, d, H-16). In the same way, compound **3** was treated with (*R*)–MTPA chloride in pyridine-*d*_5_ to give the expected (*S*)-MTPA ester derivative **3b**: ^1^H NMR (600 MHz, pyridine-*d*_5_) δ_H_ 5.971 (1H, d, H-5), 5.849 (1H, d, H-7), 1.292 (1H, m, H-8), 1.145 (1H, m, H-9a), 1.123 (1H, m, H-9b), 1.571 (3H, s, H-14), 1.875 (3H, s, H-15), 1.092 (3H, d, H-16), shown as Figures S12 and S13 and Table S1.

By the same method described above, compound **4** (0.5 mg, 1.77 μmol) was, respectively, acylated using (*S*)- and (*R*)-(-)-α-methoxy-α-(trifluoromethyl)phenylacetyl chloride (3 μL, 15.9 μmol), which resulted in products of (*R*)-MTPA ester **4a** and (*S*)-MTPA ester **4b**, respectively. **4a**: ^1^H NMR (600 MHz, pyridine-*d_5_*) δ_H_ 6.008 (1H, d, H-5), 4.057 (1H, d, H-7), 1.712 (1H, m, H-8), 1.272 (1H, m, H-9a), 1.269 (1H, m, H-9b), 1.988 (3H, s, H-14), 1.590 (3H, s, H-15), 1.206 (3H, d, H-16). **4b**: ^1^H NMR (600 MHz, pyridine-*d*_5_) δ_H_ 6.009 (1H, d, H-5), 4.058 (1H, d, H-7), 1.171 (1H, m, H-8), 1.271 (1H, m, H-9a), 1.268 (1H, m, H-9b), 1.989 (3H, s, H-14), 1.590 (3H, s, H-15), 1.206 (3H, d, H-16), shown as Figures S14 and S15 and Table S2.

### 2.5. Computational Section for Compound 3

To determine the absolute configurations of C-4 and C-8 in **3**, time-dependent density functional theory (TDDFT) method as a useful tool was applied for theoretical calculations of ECD spectra [19,20]. The conformational searches were carried out using Spartan software with the preliminary Merck Molecular Force Field (MMFF) in a 10.0 kcal mol^−1^ energy window [21]. All the obtained conformers were reoptimized at the B3LYP/6-31+G (d, p) level with the IEFPCM solvent model for methanol, and eight, twenty, nineteen and twenty conformers for **3**-(4*R*, 8*R*), **3**-(4*R,* 8*S*)*,* **3**-(4*S*, 8*R*) and **3**-(4*S*, 8*S*) with a Boltzmann population above 1% were obtained, respectively, shown as Figures S16–S19. The vibrational frequencies of these conformers were also calculated in the M06-2X/6-311++g (d, p) level, demonstrating all conformers are true minima. Then, these conformers were subjected to calculate the ECD spectra using the TDDFT method with the PBE0 functional and the def-TZVP basis set in the same solvent model, and the rotatory strength for a total of 60 exited states were considered. The Boltzmann-weighted ECD spectra from the ZPVE-corrected M06-2X/6-311++g (d, p) energies were generated in GaussView 6.0.16 software, and the results were represented in Figures S16–S19. All calculations were implemented in the Gaussian 16 package [22].

### 2.6. Antimicrobial Assay

Antimicrobial activity was investigated according to the agar dilution method described by Unemo and coworkers [23], ampicillin was used as a positive standard. Clinical strain *H. pylori* 159 was obtained from biopsy sample of gastritis patient. Isolation and identification of *H. pylori* 159 were used standard protocols on basis of colony appearance, Gram staining, and positive reactions in the rapid urease test [24]. Additionally, 10% fetal calf serum (FCS) brain heart infusion (BHI, Becton Dickinson, Sparks, NV, USA) broth or 5% FCS Columbia blood agar (Oxoid, Basingstoke, UK), supplemented with Dent selective supplement (Oxoid), were used for routinely culture of H. pylori strains. Incubation of strains were under microaerophilic conditions (10% CO_2_, 85% N_2_, and 5% O_2_ and 90% relative humidity) using a double-gas CO_2_ incubator (Binder, model CB160, Tuttlingen, Germany) at 37 °C for 48 to 72 h. Three replicates were performed for every antimicrobial assay.

Anti-*H. pylori* activities were carried out according to broth microdilution assay [25]. *H. pylori* cultures in the exponential phase of growth were diluted ten times in BHI broth and inoculated into each well containing 100 μL test compounds. The final concentration of *H. pylori* was 5 × 10^5^ to 1 × 10^6^ CFU/mL. After incubated in a microaerophilic atmos-phere at 37 °C for 3 days, the plates were examined visually. Antimicrobial activity testing of pure compound followed Antimicrobial Susceptibility Testing Standards outlined by the Clinical and Laboratory Standards Institute (CLSI) document M07-A7 (Clinical and Laboratory Standards Institute 2008) against strain *H. pylori* 159. MIC value indicated the minimum inhibitory concentration for each compound.

## 3. Results

### 3.1. Structure Elucidation

By careful comparison of the ^1^H and ^13^C NMR and ESI-MS spectral data with literature (Table 1, Figures S1 and S2), the chemical structures of compounds **3** and **4** were, respectively, identified as neovasifuranone A and neovasifuranone B [12,13,14,15], while compounds **1**, **2** and **5** were, respectively, characterized as *N*-(2-phenylethyl)acetamide [26], 1-(3-hydroxy-2-methoxyphenyl)-ethanone [27] and 1,2-seco-trypacidin [28].

**Figure 2:**
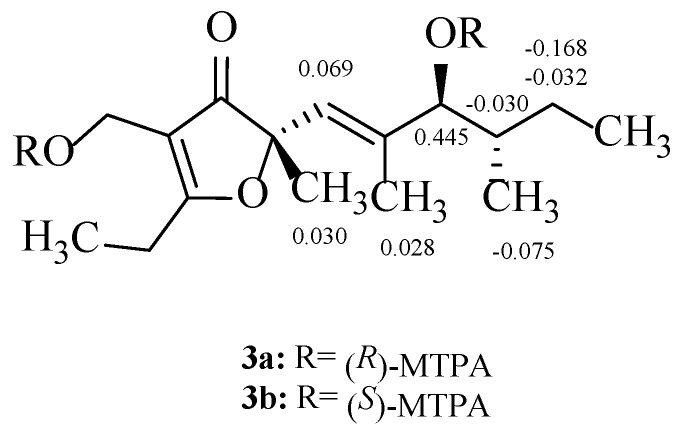
Δδ*_S_*_–*R*_ values for MTPA esters of compounds **3a** and **3b**.

**Figure 3:**
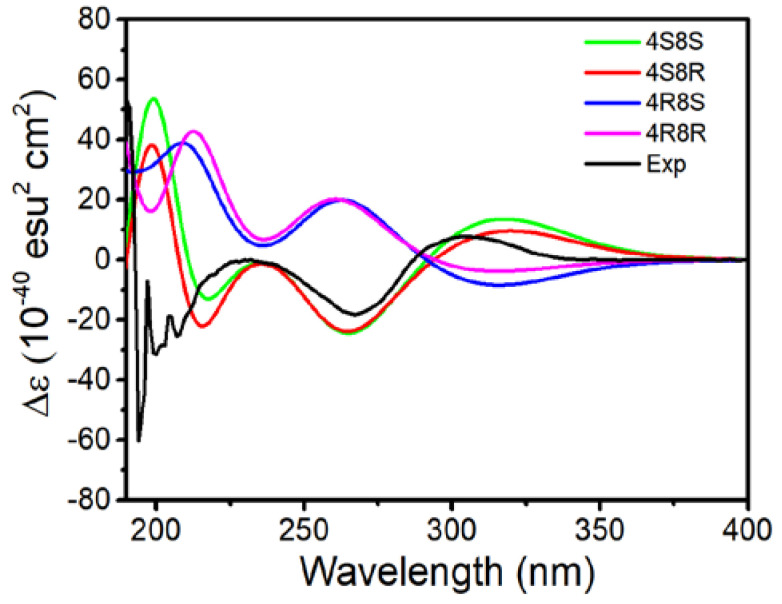
Calculated and experimental ECD spectra of compound **3**.

The absolute configurations of compounds **3** and **4** were further confirmed by a combination of modified Mosher’s reactions and calculated electronic circular dichroism (ECD) and optical rotatory dispersion (ORD) analysis. Since the more abundant compound **3** possessed two readily acylable hydroxyl groups at C-7 and C-13, its (*S*)- and (*R*)-MTPA esters (**3a** and **3b**) were prepared as detailed elsewhere [29,30,31]. The chemical shift deviations (Δ*δ_S_*_–*R*_, Figure 2) calculated from the ^1^H NMR spectral data of **3a** and **3b** indicated the presence of a 7*S*-configuration. Obviously, the calculated ECD spectra for **3**-(4*S*, 8*R*) and **3**-(4*S*, 8*S*) are similar to their experimental ECD spectra (Figure 3). Furthermore, computed ORD values for **3**-(4*S*, 8*R*) and **3**-(4*S*, 8*S*) under the 589.3 nm are, respectively, −163° and −39°, whereas the experimental ORD value for **3** is −140°, which closely agrees with the calculated ORD value of **3**-(4*S*, 8*R*) [32,33]. Therefore, the absolute configuration of **3** is unambiguously established as (4*S*, 7S, 8*R*).

**Figure 4:**
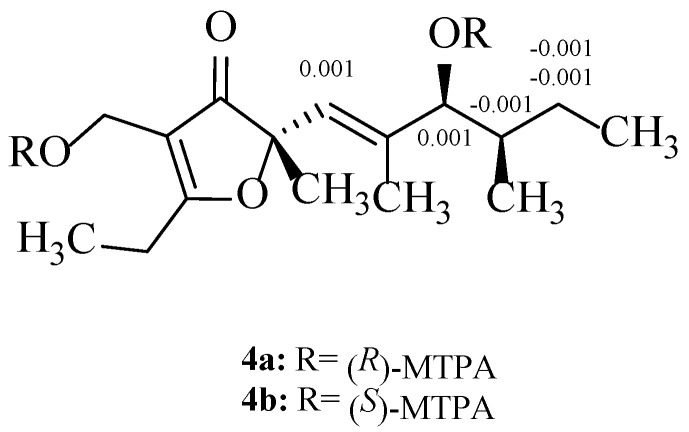
Δδ*_S_*_–*R*_ values for MTPA esters of compounds **4a** and **4b**.

**Figure 5:**
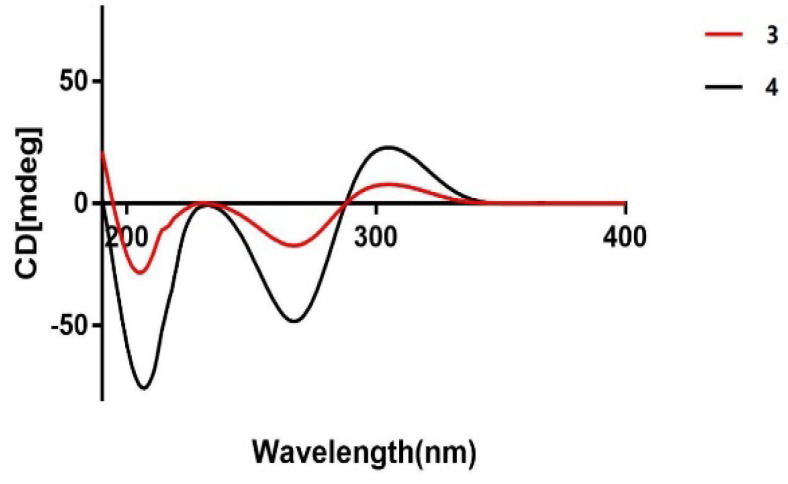
Experimental ECD spectra of compounds **3** and **4**.

As far as compound **4** concerned, the Mosher’s reaction result has a similar trend with compound **3**. The chemical shift deviations (Δ*δ_S_*_–*R*_, Figure 4) calculated from the ^1^H NMR spectral data of **4a** and **4b** indicated the presence of a 7*S*-configuration in **4**. By comparison of ECD spectrum of compounds **3** with that of **4** (Figure 5), they had very similar cotton effects, which one valley at 202 nm and a peak at 306 nm were respectively shown in the first negative and the positive cotton effect regions, and the other elliptical valley was apparent at 267 nm in the negative cotton effect region [34]. Furthermore, the experimental ORD value for **4** is −92°, which is similar to that of its isomer **3**-(4*S*, 8*S*), suggesting that three chiral centers at C-4, C-7 and C-8 in **4** are *S* configurations. Accordingly, the absolute configuration of **4** is undoubtedly characterized as (4*S*, 7*S*, 8*S*).

### 3.2. Antimicrobial Activity

Antimicrobial tests were carried out on one of the most serious pathogenic bacteria *Helicobacter pylori* 159. The results indicated that none of these compounds **1**–**5** had remarkable inhibitory effect on *H. pylori* 159, which MIC values are no less than 16 μg/mL (Table 2).

## 4. Discussion

To the best of our knowledge, more than 50% of the currently used drugs are chiral compounds. The enantiomers of the same drug have the same physical and chemical properties, but they exhibit differences in pharmacokinetics, pharmacodynamics and toxicity [35]. Owing to inherent structural and stereochemical complexity, fungal secondary metabolites play a significant role in drug discovery and development processes. In this study, the absolute configurations of two flexible molecules neovasifuranones A (**3**) and B (**4**) from *F. oxysporum* R1 were firstly determined by a combination of Mosher’s reactions and quantum mechanical calculation of chiroptical (ECD and ORD) properties. These findings will assist in further analysis of structure-activity relationship of compounds **3** and **4**.

Endophytic fungi are one of important sources of bioactive secondary metabolites with potential application in biomedicine and agriculture [36,37]. *Fusarium* microorganisms are ubiquitous in nature including terrestrial and marine environments and plants. Genome sequencing and analysis indicate that these microbes possess a great number of secondary metabolites biosynthetic gene clusters (BGCs), including polyketide synthetase, non-ribosomal peptide synthetase and terpene synthetase [9]. However, most of these cryptic BGCs are not expressed under conventional culture conditions, which result in an unfavorable trend that the number of novel natural products from the genus *Fusarium* has been decreasing in the past decade. Therefore, more efforts should be made to awaken their silent BGCs to produce novel functional biomolecules using OSMAC strategy and advanced interdisciplinary technology, such as genome mining, metabonomics, gene heteroexpression and functional characterization [38,39].

## Figures and Tables

**Table 1:** NMR spectral data for compounds **3** and **4** (^1^H, 600 MHz and ^13^C 150 MHz).

Position	Compound 3 (in DMSO-*d*_6_)		Compound 4 (in CDCl_3_)	
	*δ*_C_	*δ*_H_ (in ppm, *J* in Hz)	*δ*_C_	*δ*_H_ (in ppm, *J* in Hz)
1	189.6		190. 9	
2	112.1		112.4	
3	203.9		206.6	
4	87.7		89.1	
5	121.7	5.37 (1H, *m*)	123.2	5.42 (1H, *s*)
6	143.5		144.1	
7	78.0	3.60 (1H, *t*, *J* = 4.2)	81.4	3.70 (1H, *d*, *J* = 6.9)
7-OH		8.31 (1H, *s*)		
8	36.9	1.39 (1H, *m*)	37.5	1.50 (1H, *m*)
9	25.9	1.06 (1H, *m*)	26.4	1.05 (1H, *m*)
		1.31 (1H, *m*)		1.31 (1H, *m*)
10	11.6	0.84 (3H, *t*, *J* = 7.2)	11.9	0.87 (3H, *t*, *J* = 7.5)
11	21.9	2.62 (1H, *q*, *J* = 7.2)	22.9	2.65 (2H, *m*)
		2.67 (1H, *q*, *J* = 7.8)		
12	10.5	1.16 (3H, *t*, *J* = 7.2)	10.9	1.24 (3H, *t*, *J* = 7.5)
13	50.5	4.01 (2H, *s*)	53.1	4.24 (2H, *m*)
14	24.0	1.37 (3H, *s*)	24.5	1.47 (3H, *s*)
15	13.6	1.60 (3H, *d*, *J* = 0.6)	13.5	1.64 (3H, *d*, *J* = 1.3)
16	13.7	0.71 (3H, *d*, *J* = 6.6)	14.3	0.84 (3H, *d*, *J* = 6.7)

**Table 2:** In vitro anti-*Helicobacter pylori* effects of compounds **1**-**5**.

Compound	MIC Value (μg/mL)
	*Helicobacter pylori* 159
**1**	>16
**2**	>16
**3**	>16
**4**	>16
**5**	16
Ampicillin sodium	4

## Data Availability

Not applicable.

## Supplementary Materials

The following are available online at https://www.mdpi.com/article/10.3390/jof8010040/s1, Figure S1: (+) ESI-MS spectrum of compound **3**; Figure S2: (+) ESI-MS spectrum of **4**; Figure S3: IR (KBr) spectrum of **3**; Figure S4: IR (KBr) spectrum of **4**; Figure S5: ^1^H NMR (600 MHz, (CD_3_)_2_SO) spectrum of **3**; Figure S6: ^13^C NMR (151 MHz, (CD_3_)_2_SO) spectrum of **3**; Figure S7: ^1^H NMR (600 MHz, CDCl_3_) spectrum of **4**; Figure S8: ^13^C NMR (151 MHz, CDCl_3_) spectrum of **4**; Figure S9: DEPT-135 (CDCl_3_) spectrum of **4**; Figure S10: UV spectrum of **3** in MeOH; Figure S11: UV spectrum of **4** in MeOH; Figure S12: ^1^H NMR (600 MHz, pyridine-*d*_5_) spectrum of (*R*)-MTPA of **3**; Figure S13: ^1^H NMR (600 MHz, pyridine-*d*_5_) spectrum of (*S*)-MTPA of **3**; Figure S14: ^1^H NMR (600 MHz, pyridine-*d*_5_) spectrum of (*R*)-MTPA of **4**; Figure S15: ^1^H NMR (600 MHz, pyridine-*d*_5_) spectrum of (*S*)-MTPA of **4**; Table S1: ^1^H NMR spectral data for **3** and two MTPA esters **3a** and **3b** (^1^H, 600 MHz); Table S2: NMR spectral data for **4** and two MTPA esters **4a** and **4b** (^1^H, 600 MHz); Figure S16: Optimized conformers (≥1%) of **3**-(4*R*, 8*R*); Figure S17: Optimized conformers (≥1%) of **3**-(4*R*, 8*S*); Figure S18: Optimized conformers (≥1%) of **3**-(4*S*, 8*S*); Figure S19: Optimized conformers (≥1%) of **3**-(4*S*, 8*R*).
