# Supplementary material for: Absolute Configuration Determination of Two Diastereomeric Neovasifuranones A and B from Fusarium oxysporum R1 by a Combination of Mosher’s Method and Chiroptical Approach
Source: J Fungi (Basel). 2021 Dec 31;8(1):40. doi: 10.3390/jof8010040 (PMC8779425; doi:10.3390/jof8010040)
Supplement: Supplementary file 1 [file jof-08-00040-s001.zip › jof-1522934-supplementary.pdf]

Supplementary materials for

Absolute configuration determination of two diastereomeric polyketides neovasifuranones A and B from *Fusarium oxysporum* R1 by a combination of Mosher`s method and chiroptical approach

Zhiyang Fu [1], Yuanyuan Liu [1], Meijie Xu [1], Xiaojun Yao [2], Hong Wang [1] and Huawei Zhang [1,*]

[1] School of Pharmaceutical Sciences, Zhejiang University of Technology, Hangzhou 310014, China; fuzhiyoung@163.com

[2] Department of Chemistry, Lanzhou University, Lanzhou 730000, China

[3] Key Laboratory of Marine Fishery Resources Exploitment & Utilization of Zhejiang Province, Hangzhou 310014, China

* Correspondence: hwzhang@zjut.edu.cn; Tel.: 0086-571-88320913

Contents

Figure S1. (+) ESI-MS spectrum of compound **3** 2
Figure S2. (+) ESI-MS spectrum of compound **4** 3
Figure S3. IR (KBr) spectrum of compound **3** 4
Figure S4. IR (KBr) spectrum of compound **4** 5
Figure S5. ^{1}H NMR (600 MHz, $(CD_3)_2SO$) spectrum of compound **3** 6
Figure S6. ^{13}C NMR (151 MHz, $(CD_3)_2SO$) spectrum of compound **3** 7
Figure S7. ^{1}H NMR (600 MHz, $CDCl_3$) spectrum of compound **4** 8
Figure S8. ^{13}C NMR (151 MHz, $CDCl_3$) spectrum of compound **4** 9
Figure S9. DEPT (151 MHz, $CDCl_3$) spectrum of compound **4** 10
Figure S10. UV spectrum of compound **3** in MeOH 11
Figure S11. UV spectrum of compound **4** in MeOH 12
Figure S12. ^{1}H NMR (600 MHz, pyridine-d_5) spectrum of (*R*)-MTPA of compound **3** 13
Figure S13. ^{1}H NMR (600 MHz, pyridine-d_5) spectrum of (*S*)-MTPA of compound **3** 14
Table S1. ^{1}H NMR spectral data for **3** and its MTPA esters **3a** and **3b** (^{1}H, 600 MHz) 15
Figure S14. ^{1}H NMR (600 MHz, pyridine-d_5) spectrum of (*R*)-MTPA of compound **4** 16
Figure S15. ^{1}H NMR (600 MHz, pyridine-d_5) spectrum of (*S*)-MTPA of compound **4** 17
Table S2. ^{1}H NMR spectral data for **4** and its MTPA esters **4a** and **4b** (^{1}H, 600 MHz) 18
Figure S16. Optimized conformers (≥1%) of **3**-(4*R*, 8*R*) 19
Figure S17. Optimized conformers (≥1%) of **3**-(4*R*, 8*S*) 20
Figure S18. Optimized conformers (≥1%) of **3**-(4*S*, 8*S*) 21
Figure S19. Optimized conformers (≥1%) of **3**-(4*S*, 8*R*) 22

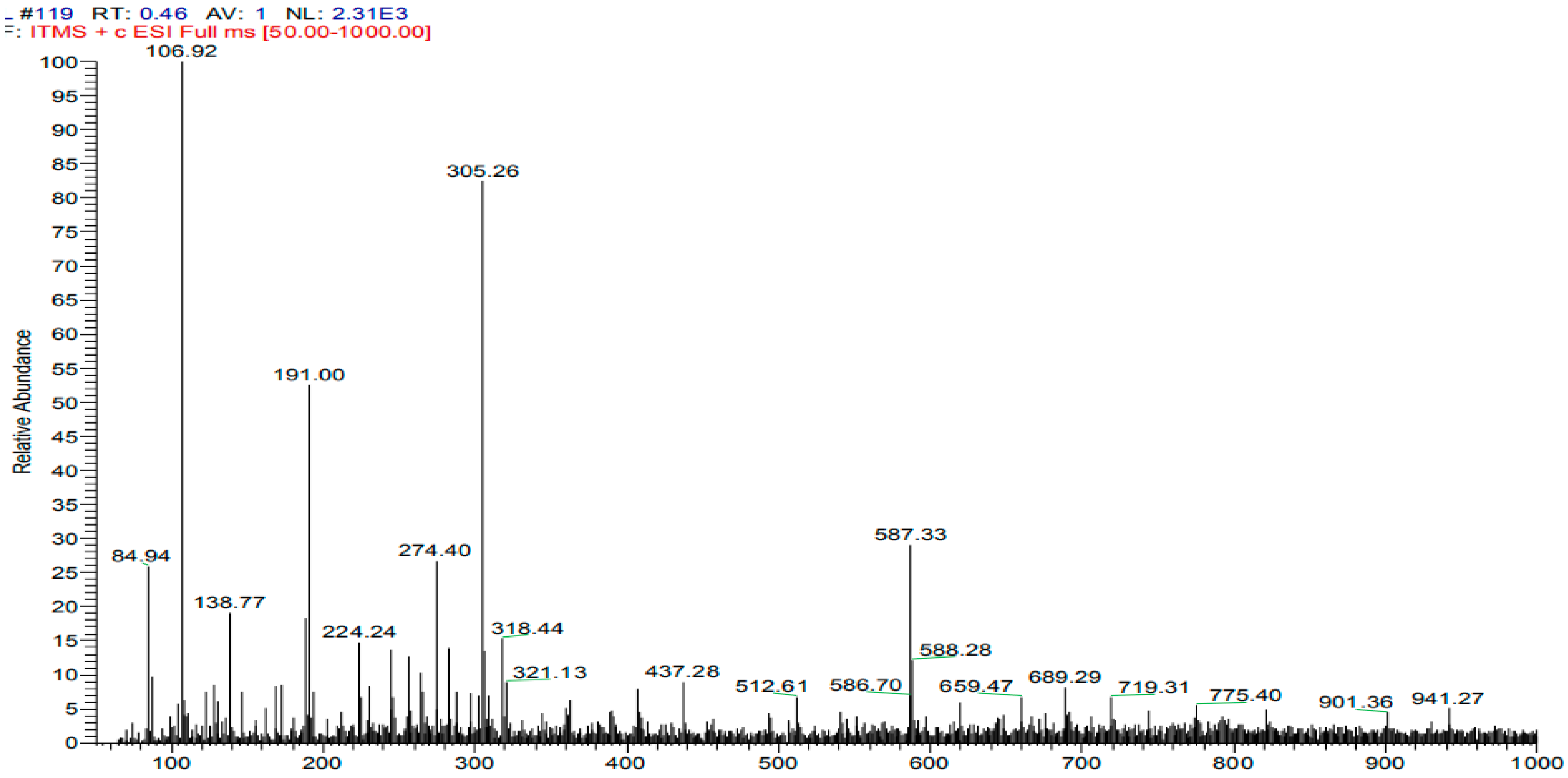

Figure S1. (+) ESI-MS spectrum of compound **3.**

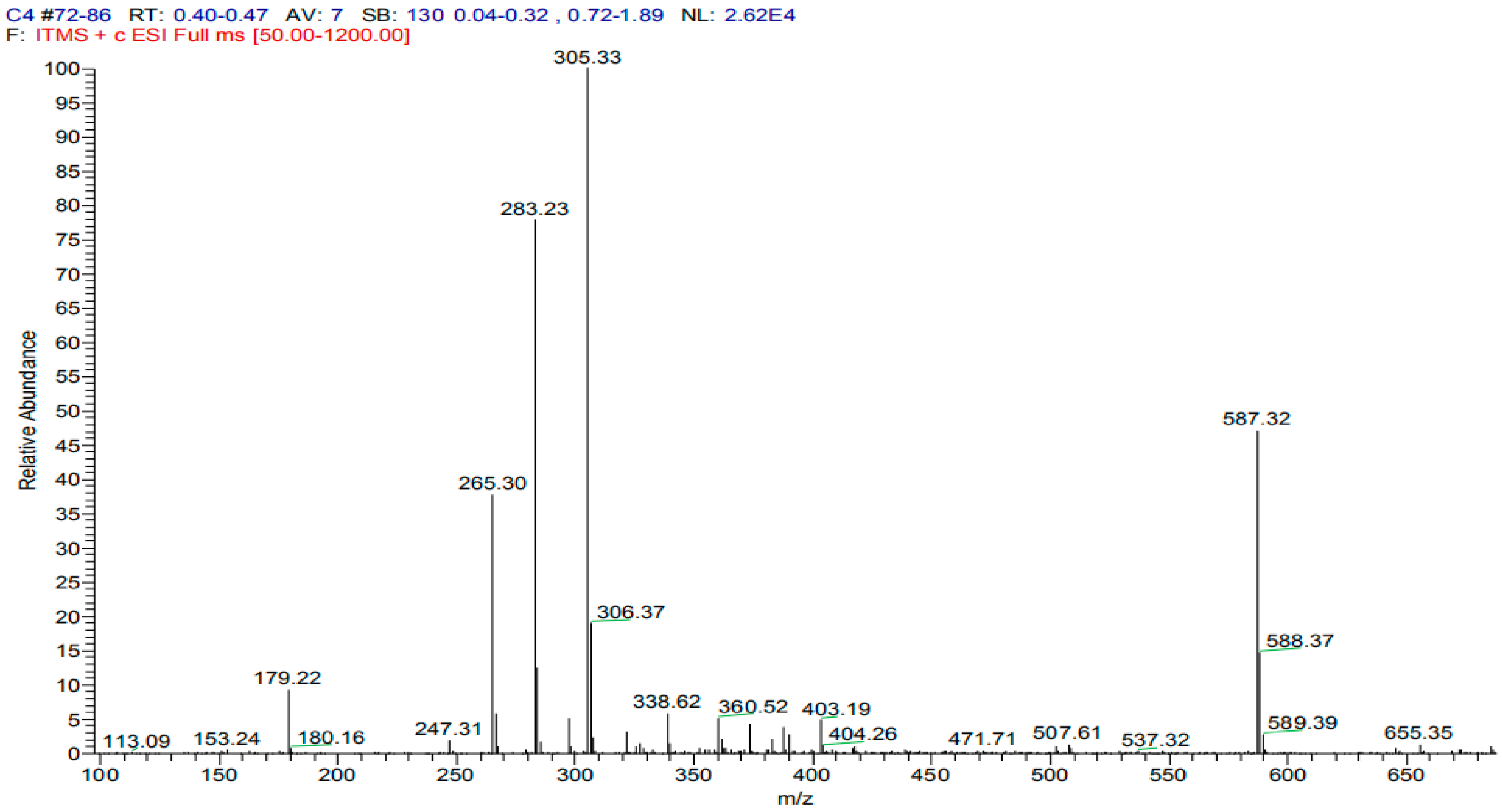

Figure S2. (+) ESI-MS spectrum of compound **4.**

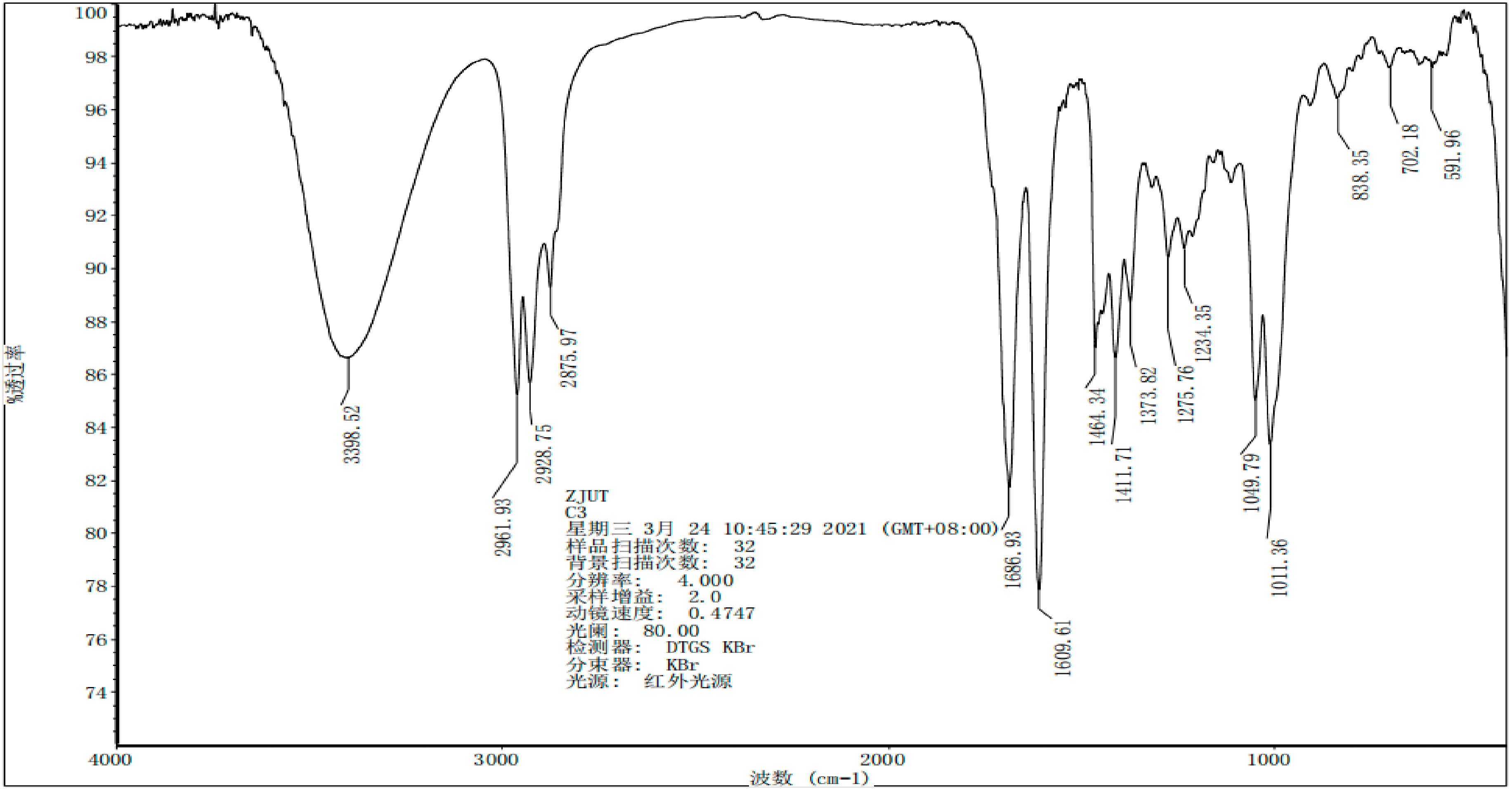

Figure S3. IR (KBr) spectrum of compound **3.**

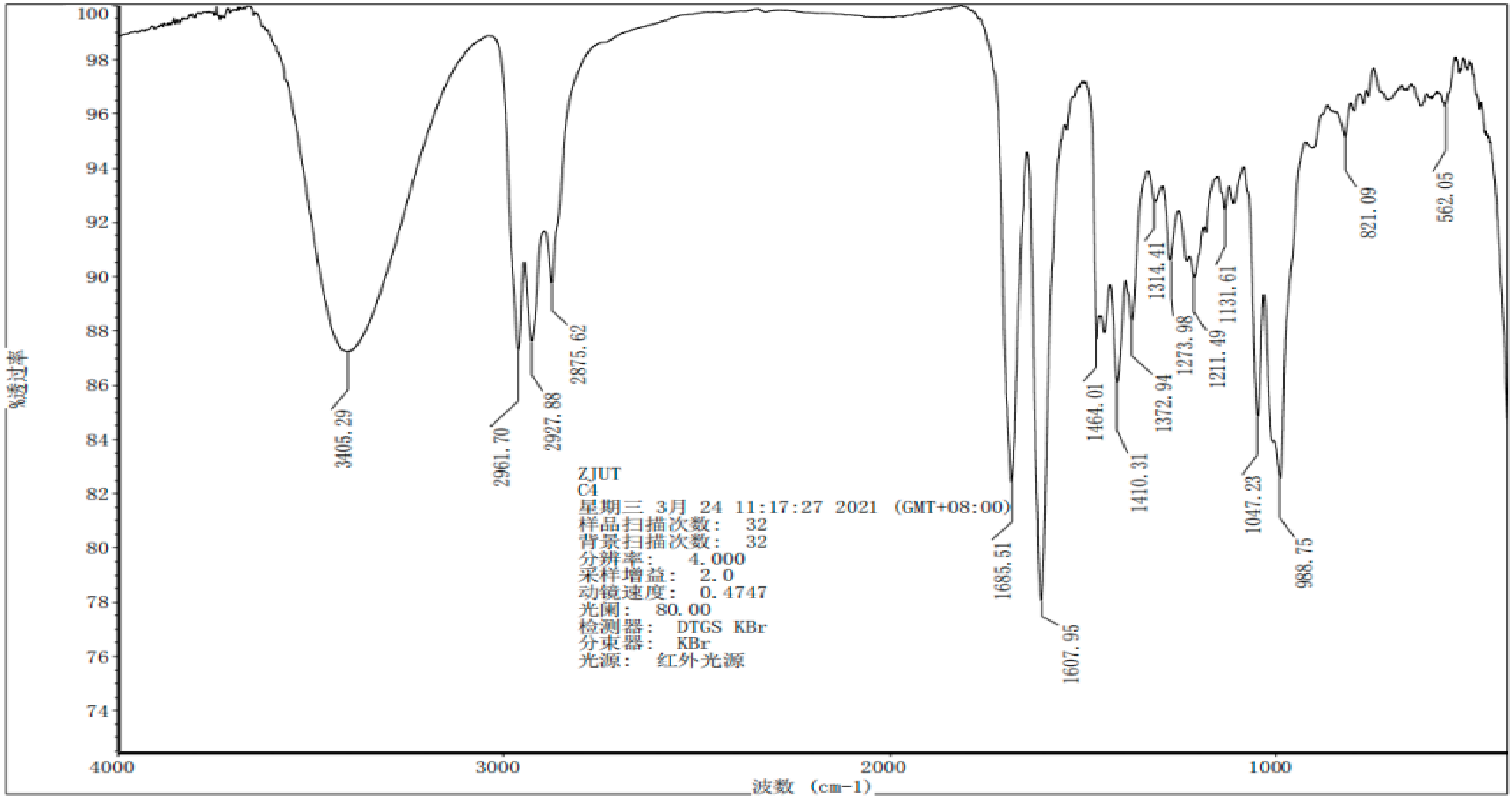

Figure S4. IR (KBr) spectrum of compound **4.**

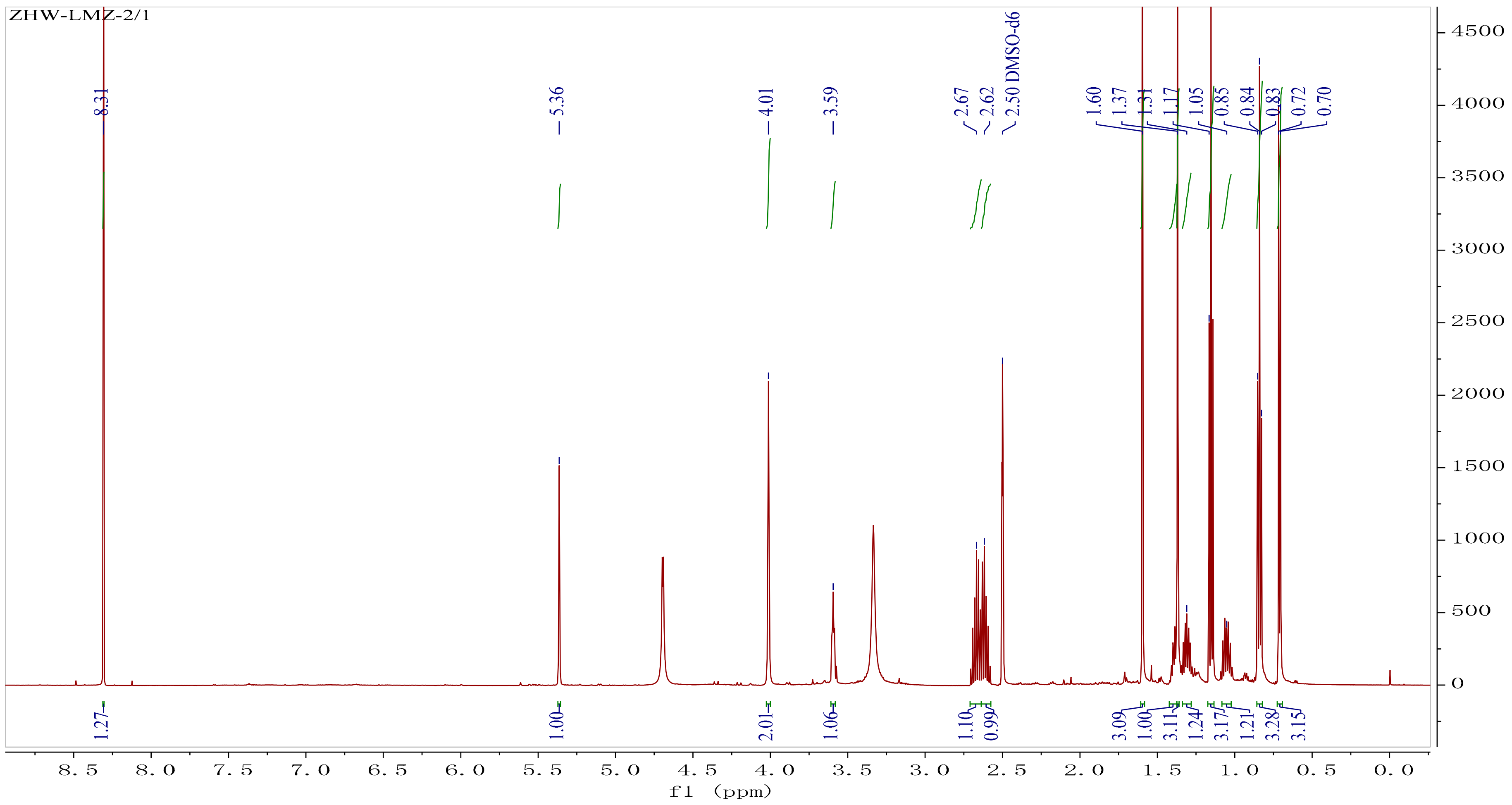

Figure S5. ^1H NMR (600 MHz, $(CD_3)_2SO$) spectrum of compound **3.**

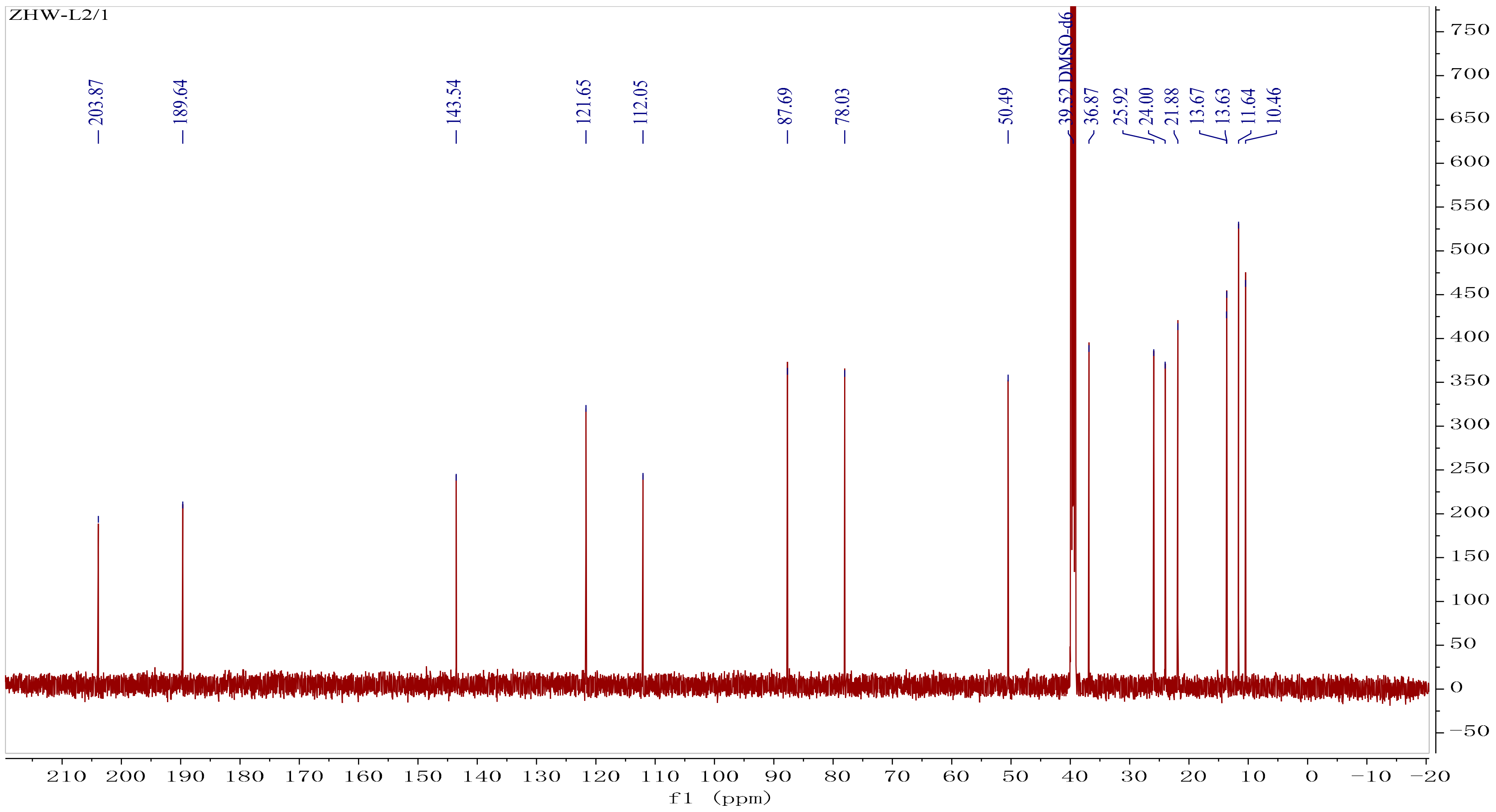

Figure S6. ^{13}C NMR (151 MHz, $(CD_3)_2SO$) spectrum of compound **3.**

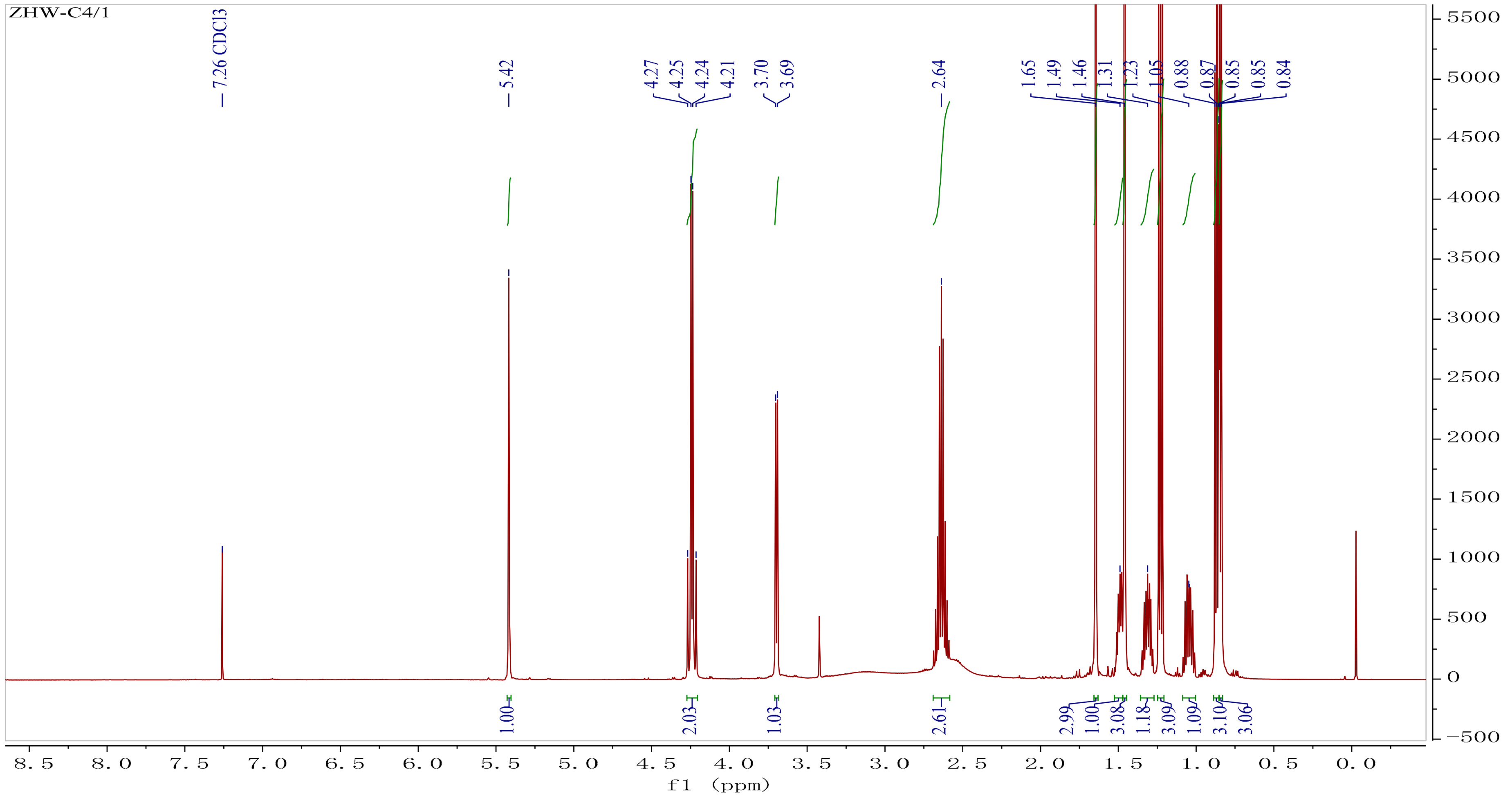

Figure S7. ^{1}H NMR (600 MHz, $CDCl_3$) spectrum of compound **4.**

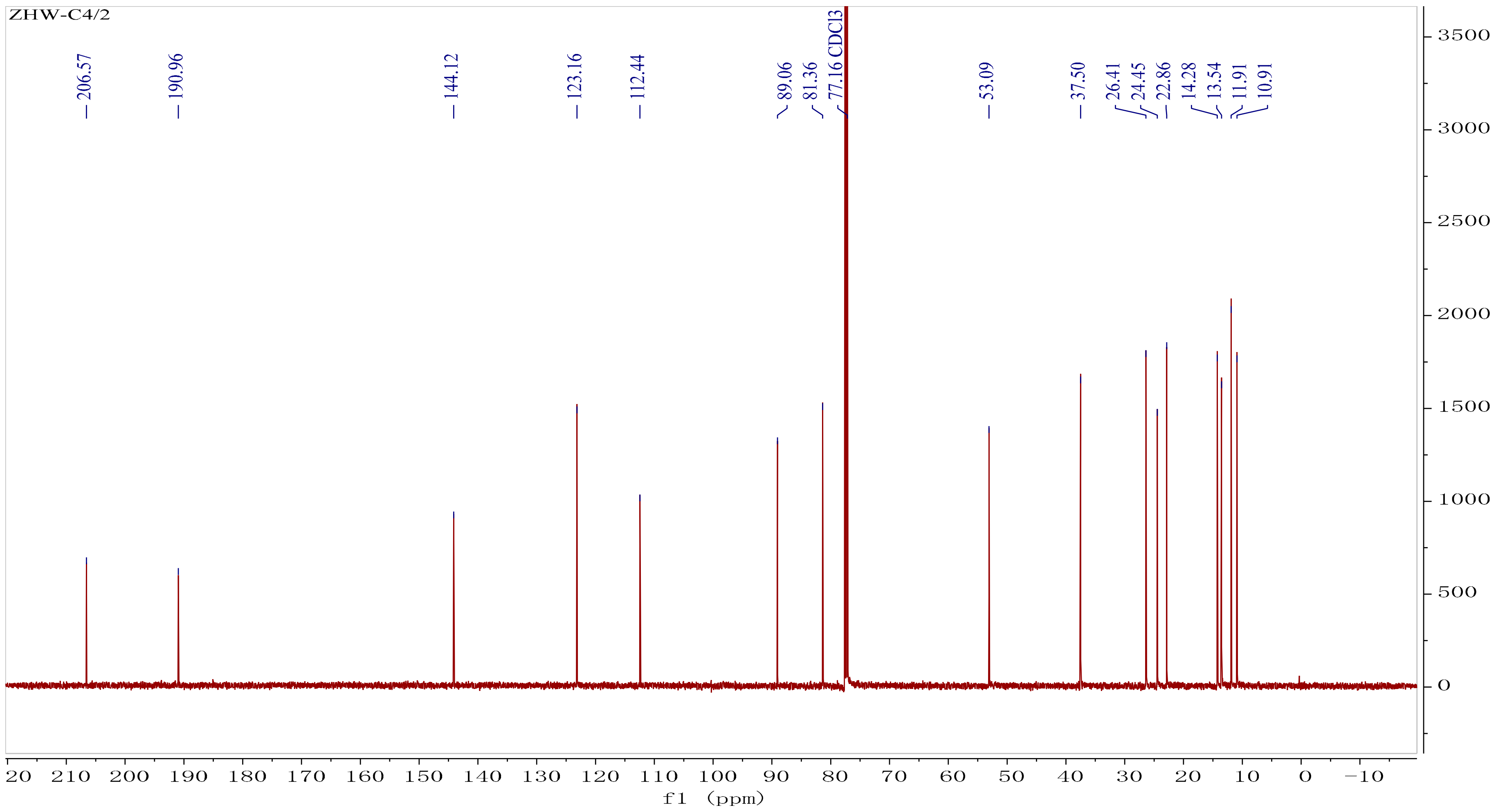

Figure S8. ^{13}C NMR (151 MHz, $CDCl_3$) spectrum of compound **4.**

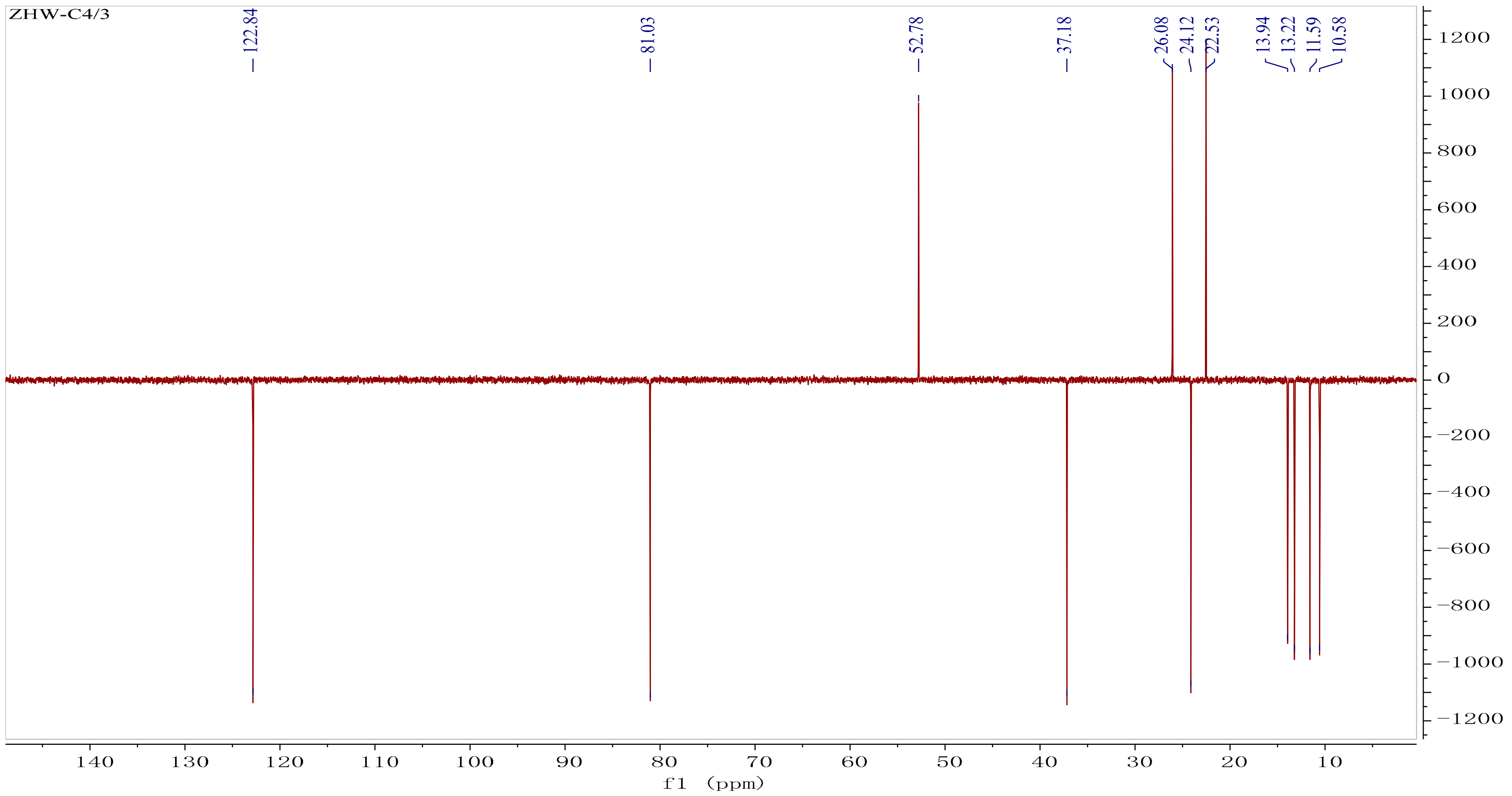

Figure S9. DEPT (151 MHz, $CDCl_3$) spectrum of compound **4.**

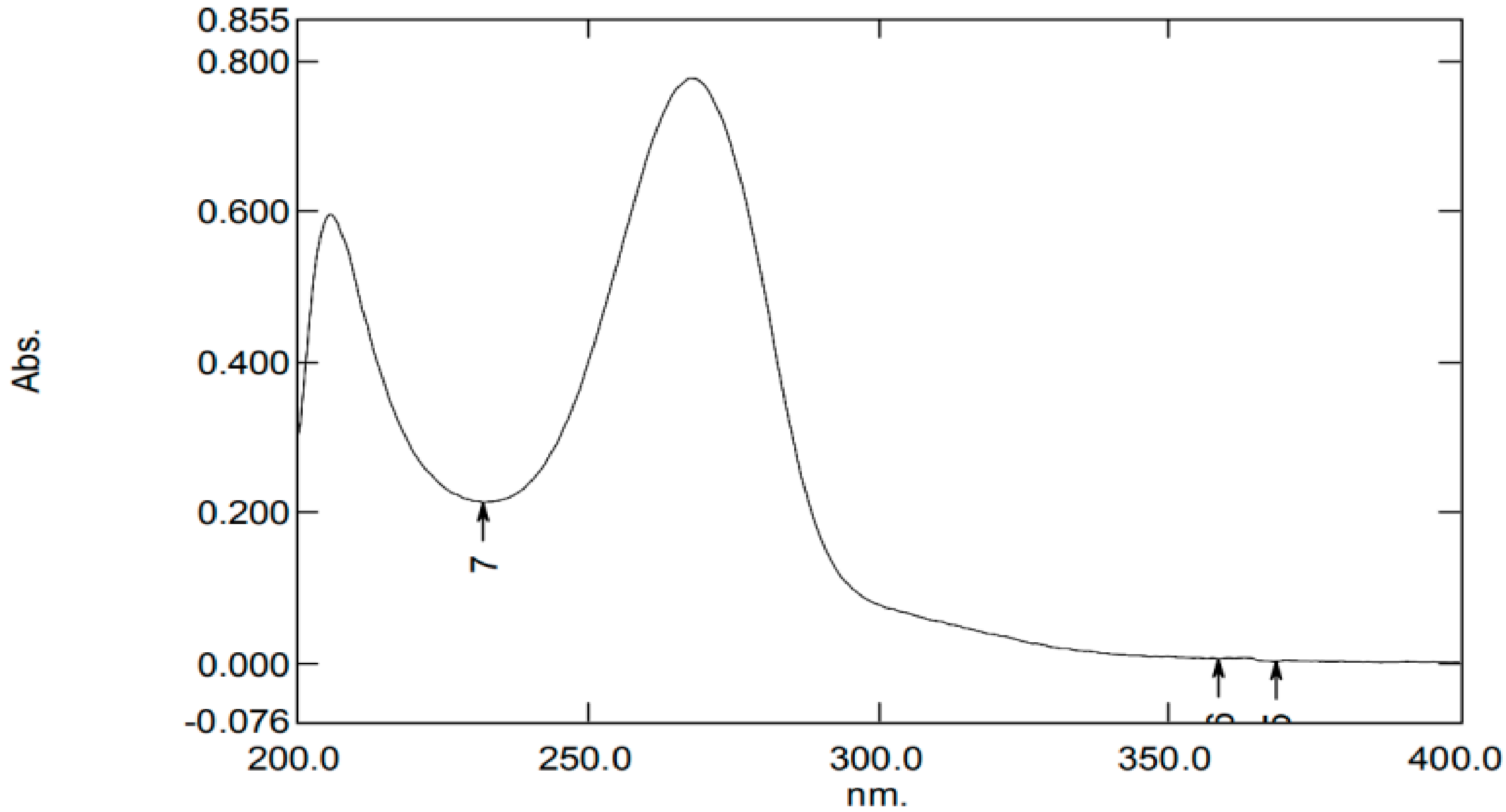

Figure S10. UV spectrum of compound **3** in MeOH.

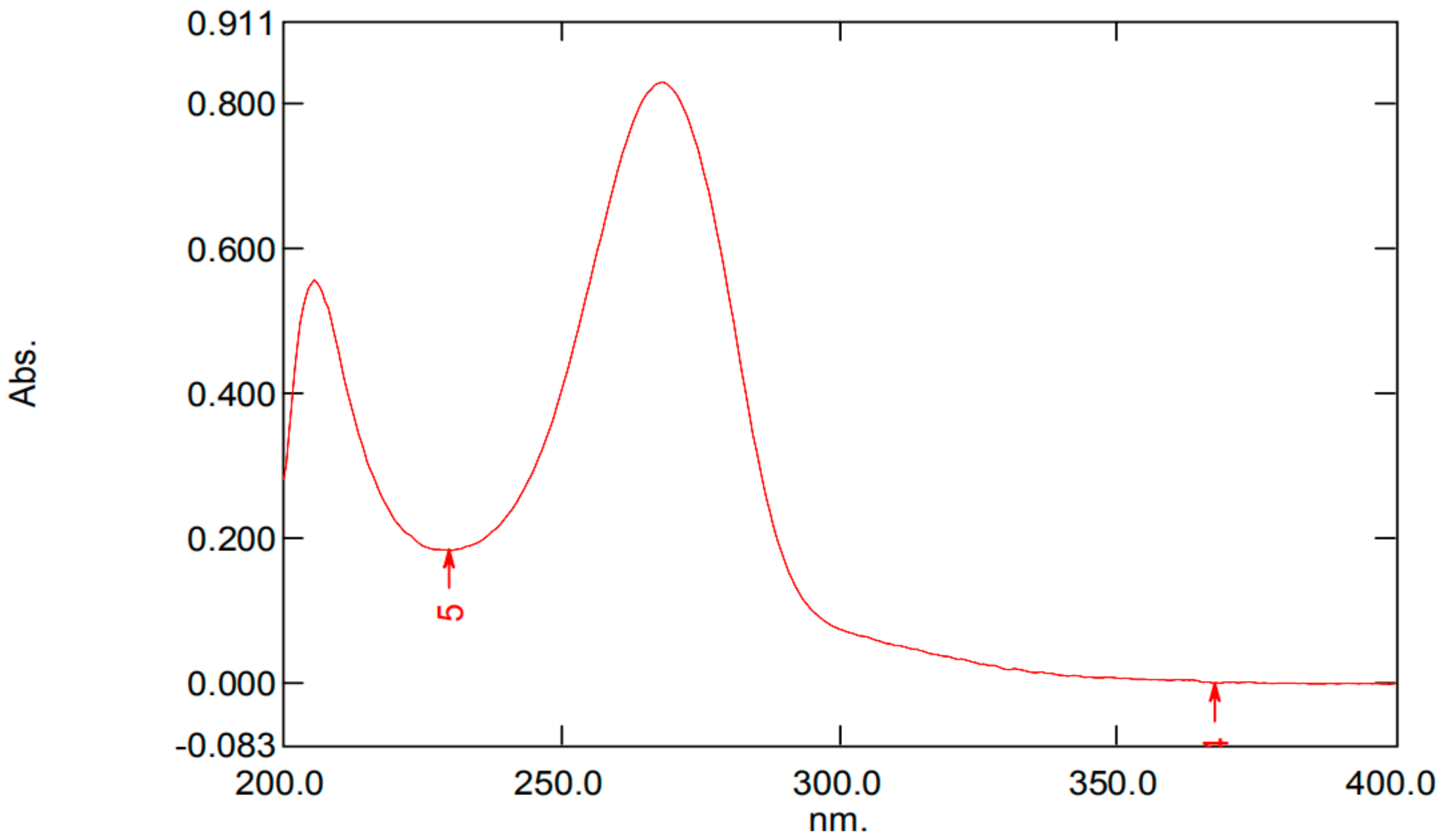

Figure S11. UV spectrum of compound **4** in MeOH.

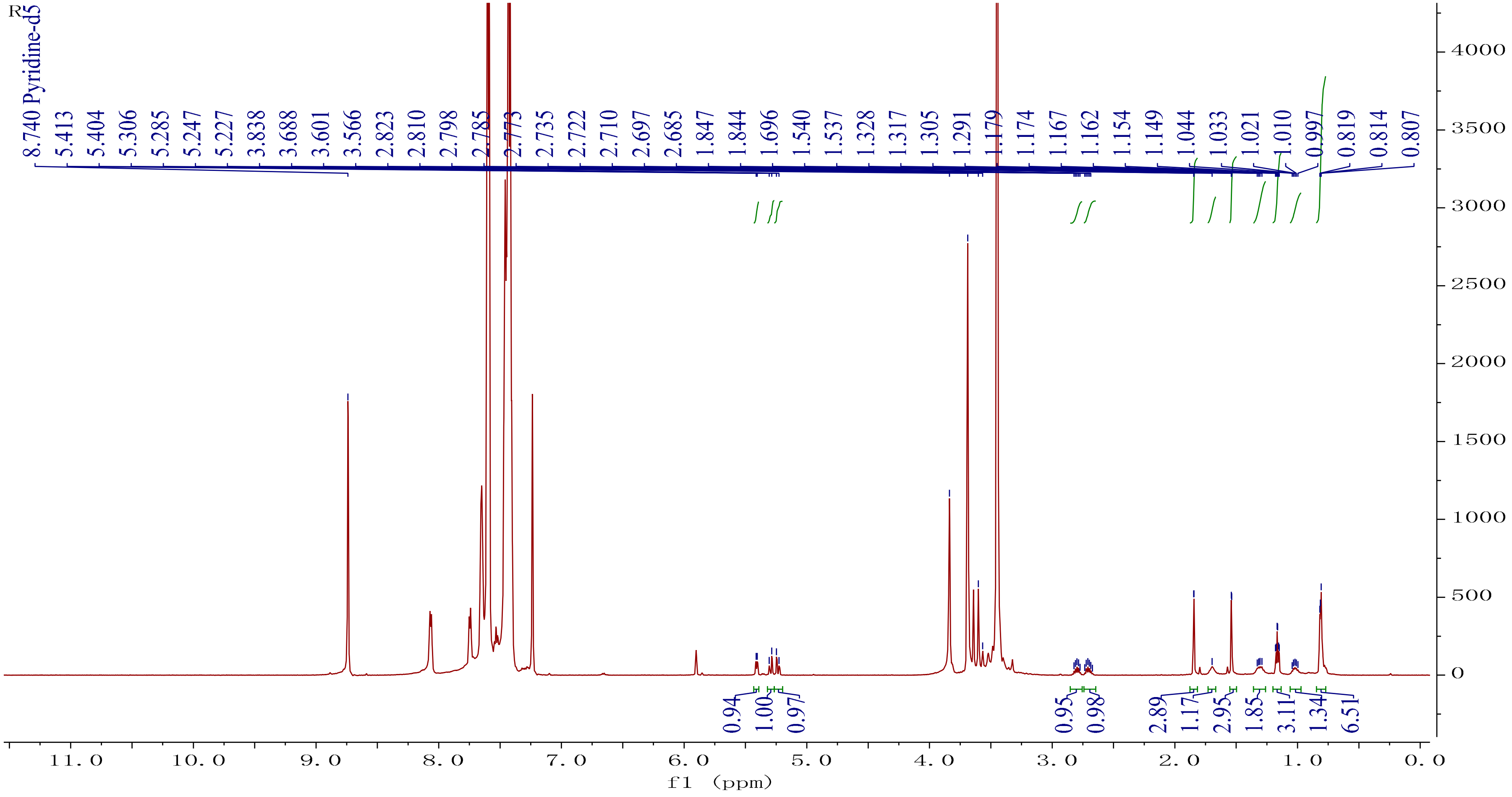

Figure S12. ^{1}H NMR (600 MHz, pyridine-d_5) spectrum of (*R*)-MTPA of compound **3.**

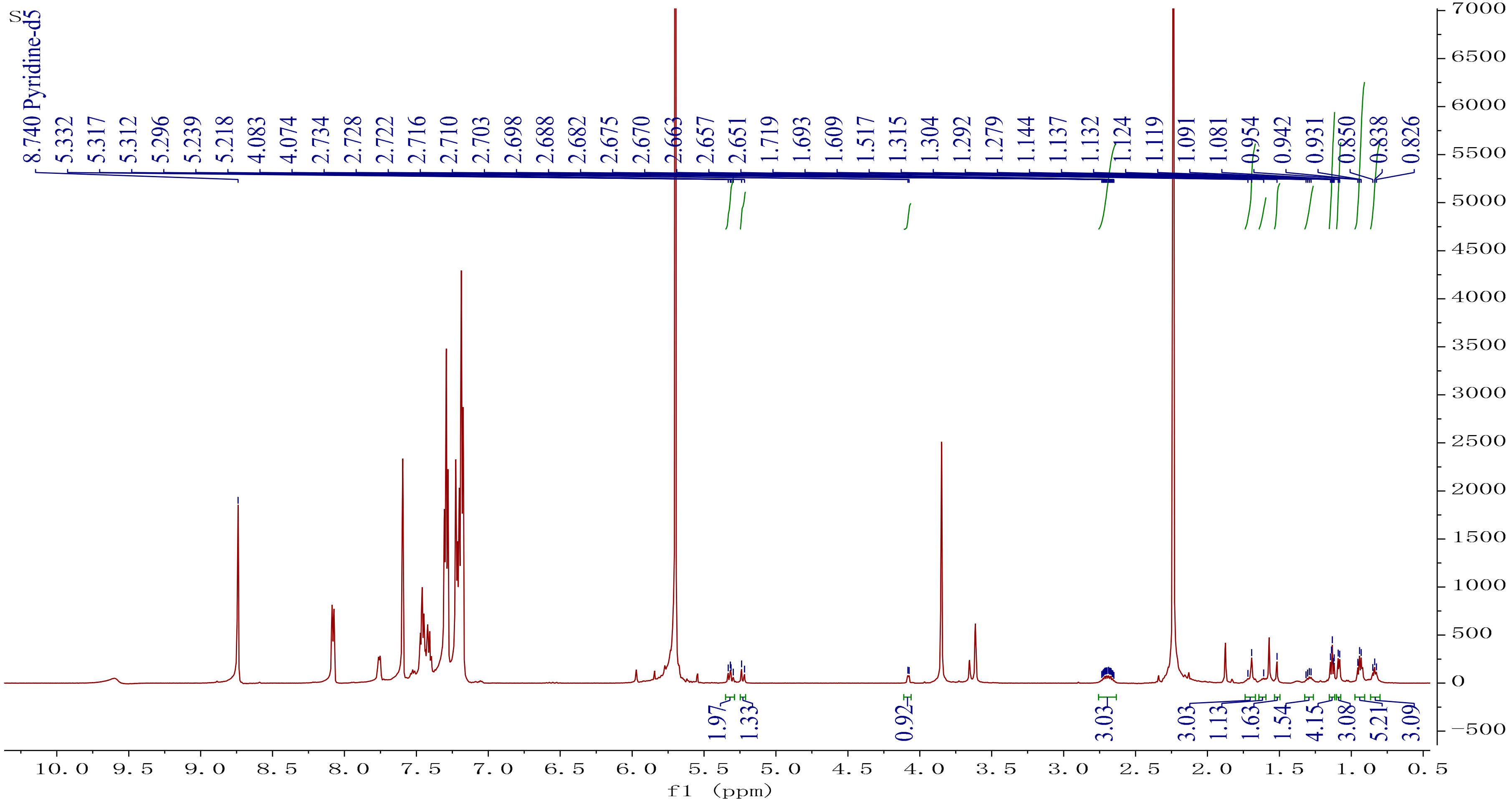

Figure S13. ^{1}H NMR (600 MHz, pyridine-d_5) spectrum of (*S*)-MTPA of compound **3**.

Table S1. ^{1}H NMR spectral data for **3** and its MTPA esters **3a** and **3b** (^{1}H, 600 MHz).

**position**	Compound **3** (in DMSO-d_6)	MTPA essters (in pyridine-d_5)	
	δ_H (in ppm, *J* in Hz)	δ_H (**3a**)	δ_H (**3b**)
1			
2			
3			
4			
5	5.37 (1H, *m*)	5.902 (1H, *m*)	5.971 (1H, *m*)
6			
7	3.60 (1H, *t*, *J* = 4.2)	5.404 (1H, *t*, *J* = 5.6)	5.849(1H, *t*, *J* = 3.1)
7-OH	8.31 (1H, *s*)		
8	1.39 (1H, *m*)	1.322 (1H, *m*)	1.292 (1H, *m*)
9	1.06 (1H, *m*)	1.177 (1H, *m*)	1.145 (1H, *m*)
	1.31 (1H, *m*)	1.292 (1H, *m*)	1.123 (1H, *m*)
10	0.84 (3H, *t*, *J* = 7.2)	1.154 (3H, *t*, *J* = 7.5)	1.137 (3H, *t*, *J* = 7.6)
11	2.62 (1H, *q*, *J* = 7.2)	2.798 (1H, *q*, *J* = 7.3)	2.703 (1H, *q*, *J* = 7.4)
	2.67 (1H, *q*, *J* = 7.8)	2.710 (1H, *q*, *J* = 7.4)	2.688 (1H, *q*, *J* = 7.3)
12	1.16 (3H, *t*, *J* = 7.2)	0.807 (1H, *q*, *J* = 6.8)	0.942 (1H, *q*, *J* = 6.8)
13	4.01 (2H, *s*)	5.306 (2H, *s*)	5.296 (2H, *s*)
14	1.37 (3H, *s*)	1.541 (3H, *s*)	1.571 (3H,*s*)
15	1.60 (3H, *d*, *J* = 0.6)	1.847 (3H, *d*, *J* = 1.7)	1.875 (3H, *d*, *J* = 1.3)
16	0.71 (3H, *d*, *J* = 6.6)	1.167 (3H, *d*, *J* = 6.7)	1.092 (3H, *d*, *J* = 6.5)

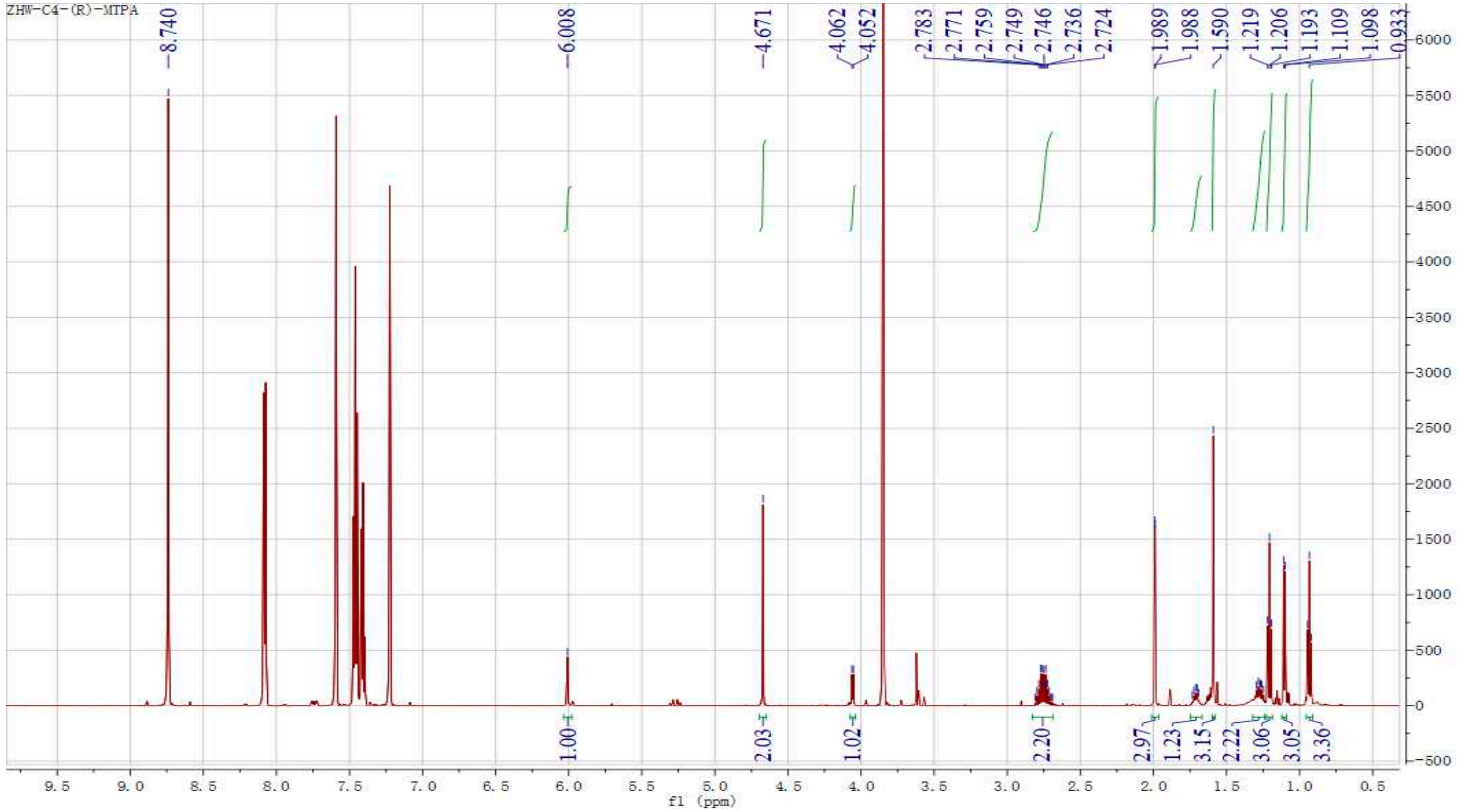

Figure S14. ^{1}H NMR (600 MHz, pyridine-d_5) spectrum of (*R*)-MTPA of compound **4.**

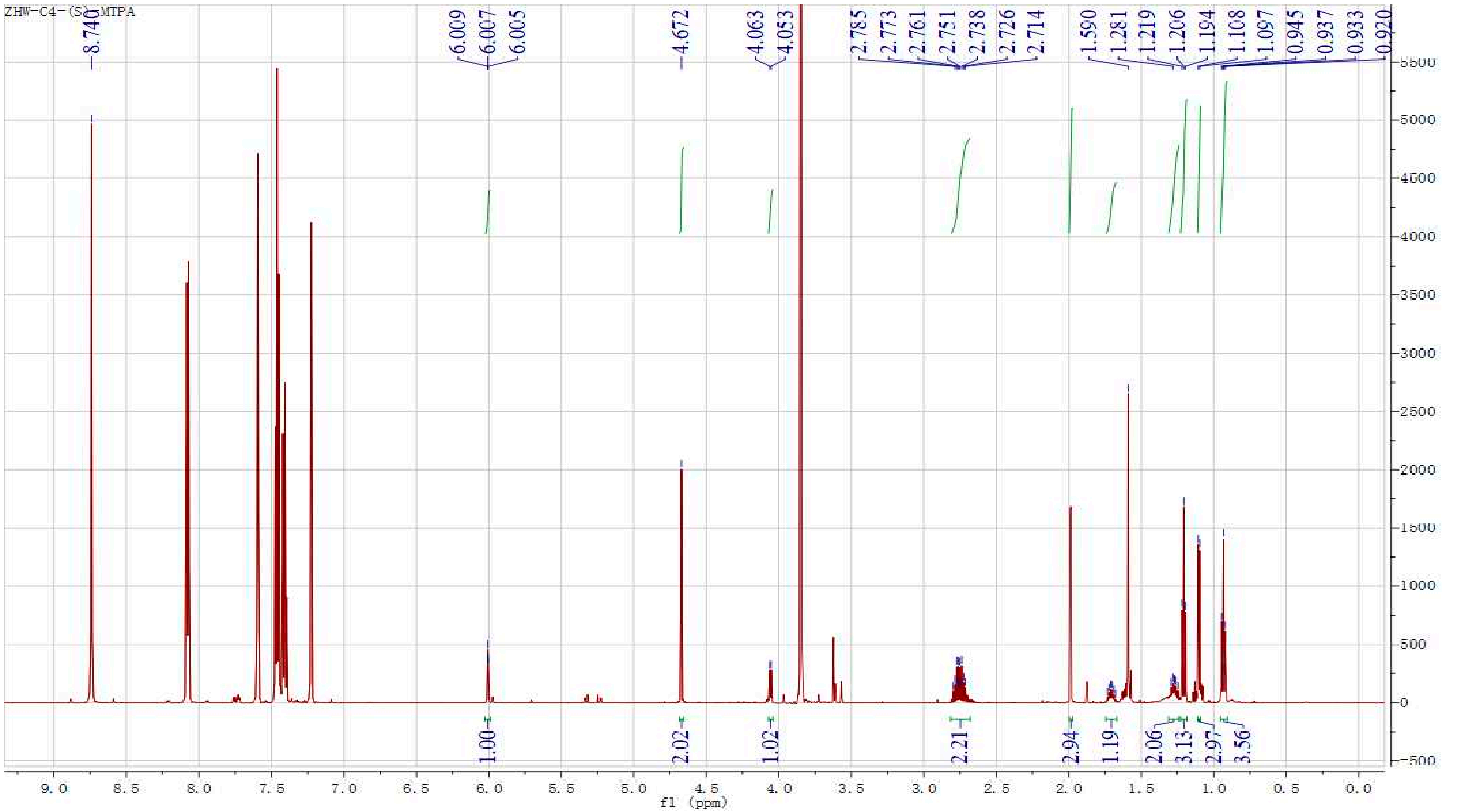

Figure S15. ^{1}H NMR (600 MHz, pyridine-d_5) spectrum of (*S*)-MTPA of compound **4.**

Table S2. ^{1}H-NMR spectral data for **4** and its MTPA esters **4a** and **4b** (^{1}H, 600 MHz).

position	Compound **4** (in $CDCl_3$)	**4a** (in pyridine-*d*$_5$)	**4b** (in pyridine-*d*$_5$)
	δ_H (in ppm, J in Hz)	δ_H (in ppm, J in Hz)	δ_H (in ppm, J in Hz)
1			
2			
3			
4			
5	5.42 (1H, s)	6.008 (1H, s)	6.009 (1H, s)
6			
7	3.70 (1H, d, J = 6.9)	4.507 (1H, d, J = 5.9)	4.508(1H, d, J = 5.8)
7-OH			
8	1.50 (1H, m)	1.712 (1H, m)	1.711 (1H, m)
9	1.05 (1H, m)	1.272 (1H, m)	1.271 (1H, m)
	1.31 (1H, m)	1.269 (1H, m)	1.268 (1H, m)
10	0.87 (3H, t, J = 7.5)	0.933 (3H, t, J = 7.3)	0.933 (3H, t, J = 7.4)
11	2.65 (2H, m)	2.755 (2H, m)	2.756 (2H, m)
12	1.24 (3H, t, J = 7.5)	1.108 (3H, t, J = 6.7)	1.109 (3H, t, J = 6.7)
13	4.24 (2H, m)	4.671 (2H, m)	4.672 (2H, m)
14	1.47 (3H, s)	1.988 (3H, s)	1.989 (3H, s)
15	1.64 (3H, d, J = 1.3)	1.590 (3H)	1.590 (3H)
16	0.84 (3H, d, J = 6.7)	1.206 (3H, d, J = 7.6)	1.206 (3H, d, J = 7.6)

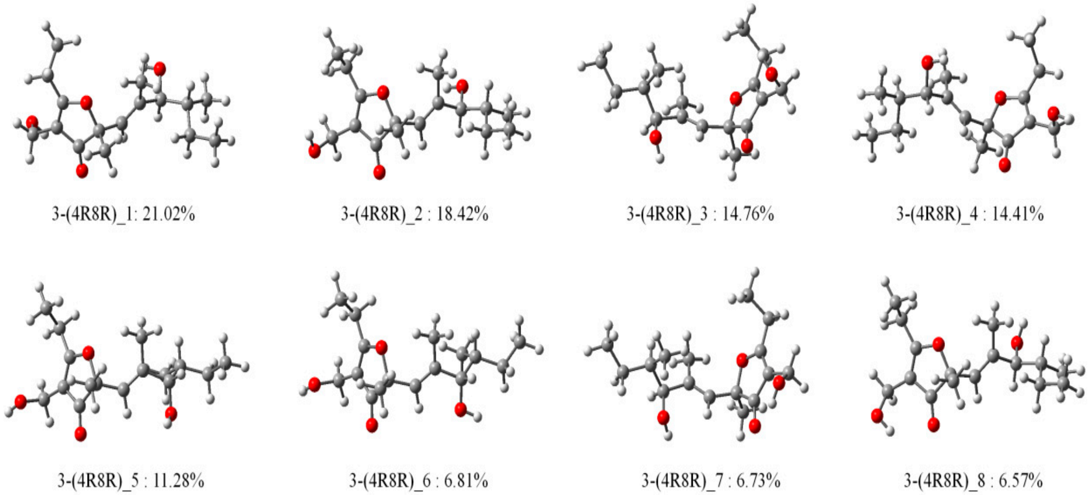

Figure S16. Optimized conformers (≥1%) of **3**-(4*R*, 8*R*).

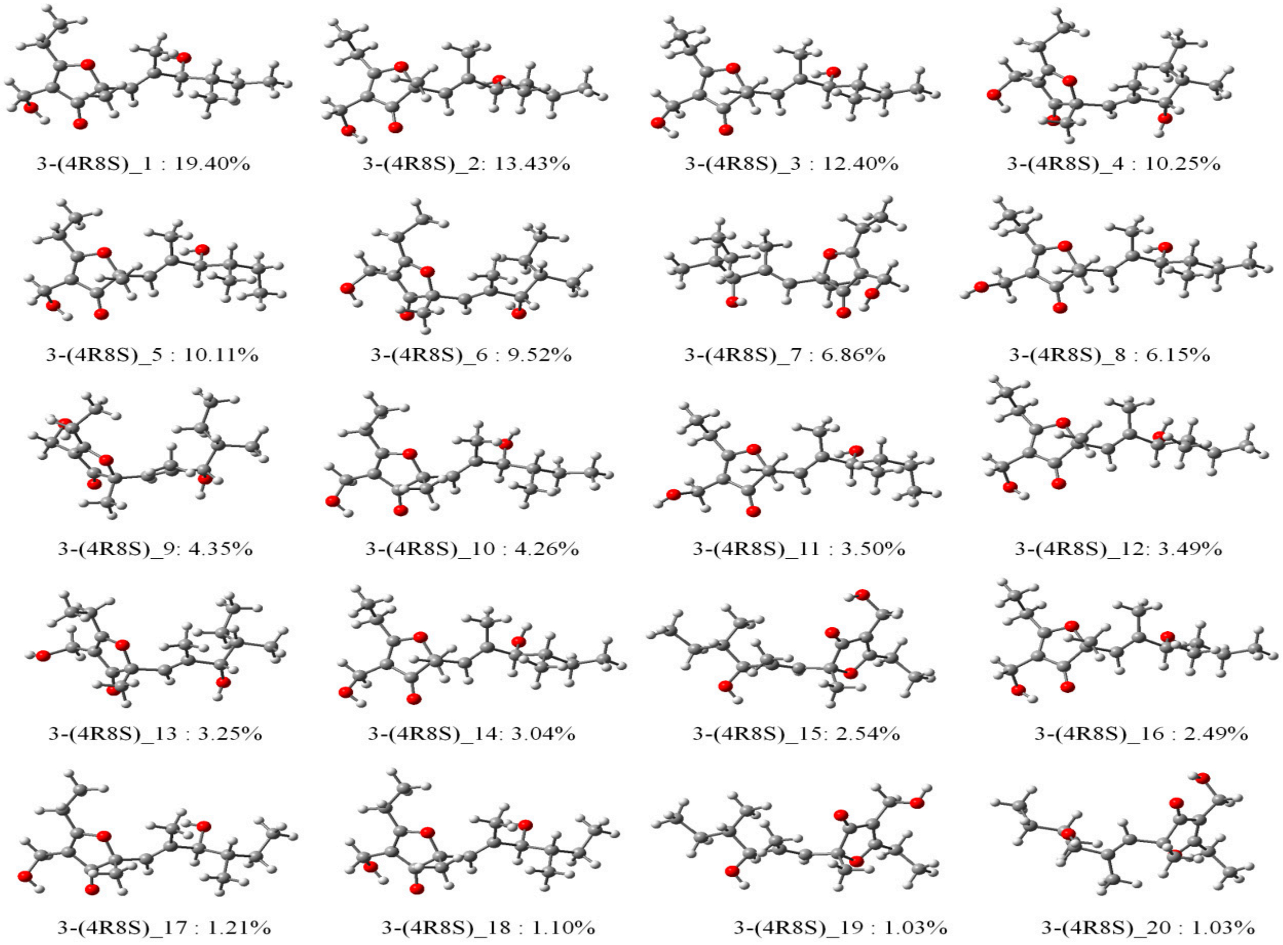

Figure S17. Optimized conformers (≥1%) of **3**-(4*R*, 8*S*).

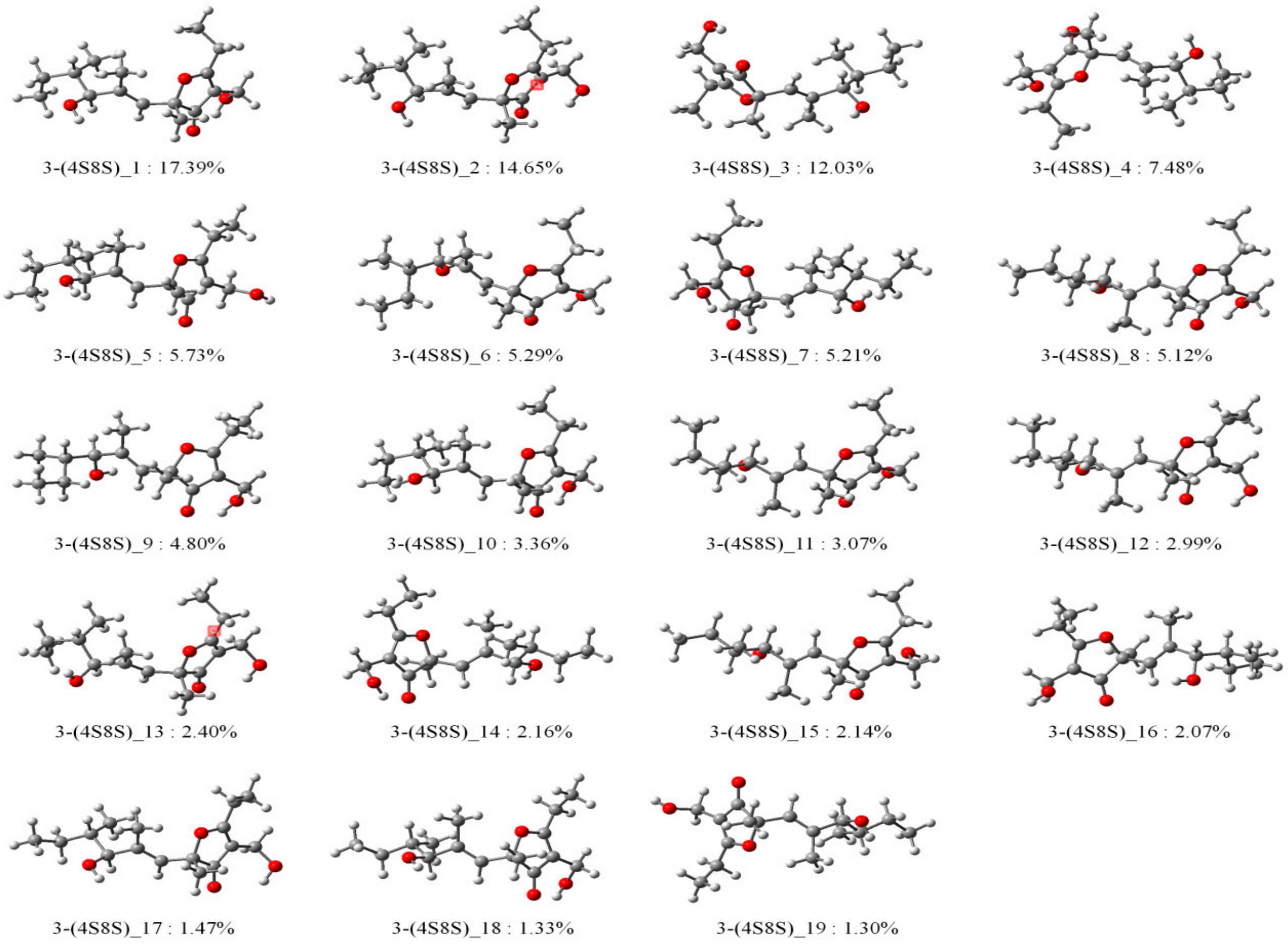

Figure S18. Optimized conformers (≥1%) of **3**-(4*S*, 8*S*).

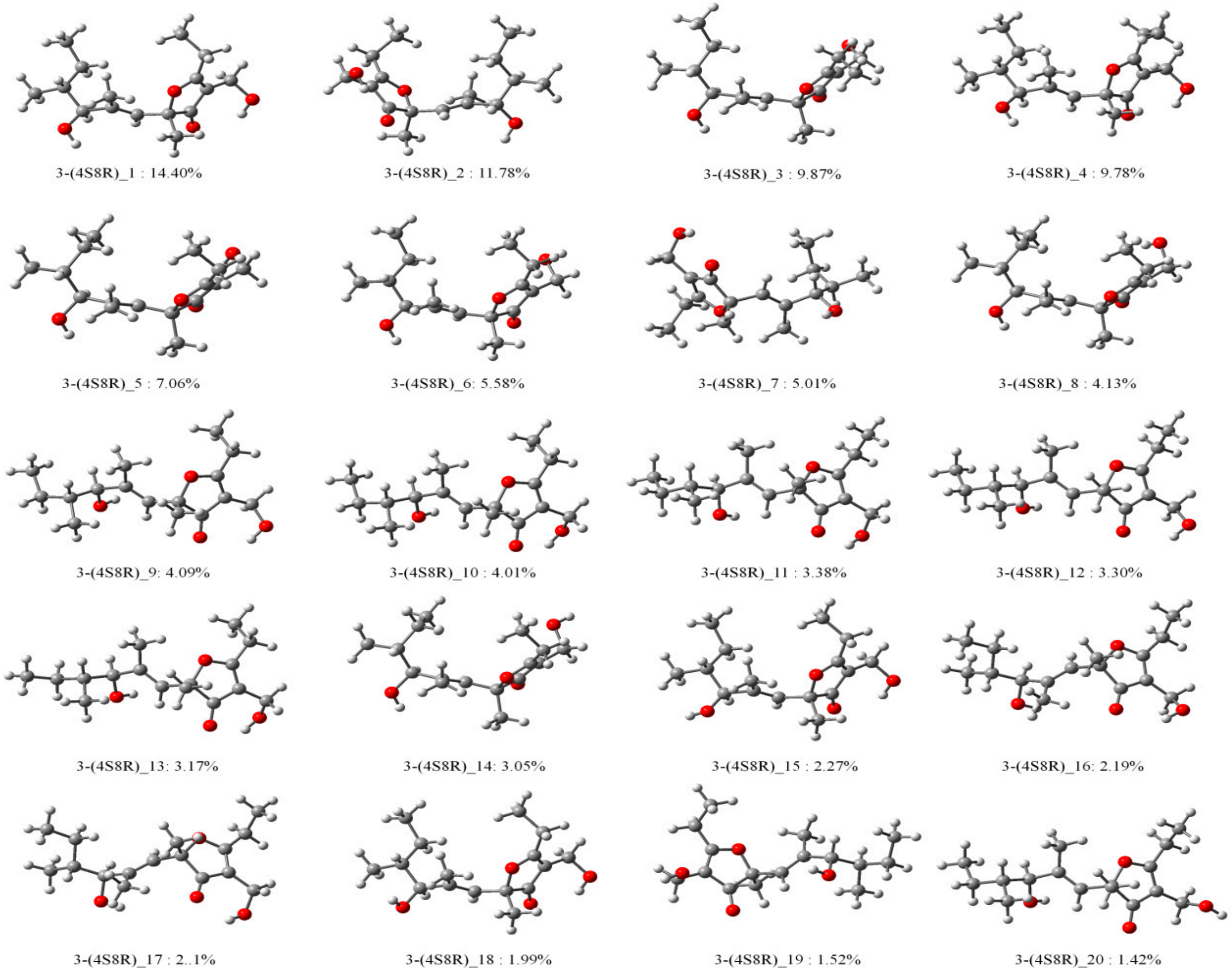

Figure S19. Optimized conformers (≥1%) of **3**-(4*S*, 8*R*).
